# Supplementary material for: Involvement of Acquired Tobramycin Resistance in the Shift to the Viable but Non-Culturable State in Pseudomonas aeruginosa
Source: Int J Mol Sci. 2023 Jul 18;24(14):11618. doi: 10.3390/ijms241411618 (PMC10380639; doi:10.3390/ijms241411618)
Supplement: Supplementary file 1 [file ijms-24-11618-s001.zip › Supplementary Table 1 Mangiaterra et al., IJMS 2023.pdf]

Supplementary Table S1. Tobramycin (TOB) and Carbonyl Cyanide 3-Chlorophenylhydrazone (CCCP) Minimum Inhibitory Concentrations (MICs) against the *P. aeruginosa* strains used in this study

	MIC		
***P. aeruginosa* strain**	**TOB (μg/ml)**	**CCCP (μM)**	**TOB+CCCP 100 μM (μg/ml)**
PAO1-N	1	>400	2
C30	16	400	2
AR86	256	400	512
