# Supplementary material for: Involvement of Acquired Tobramycin Resistance in the Shift to the Viable but Non-Culturable State in Pseudomonas aeruginosa
Source: Int J Mol Sci. 2023 Jul 18;24(14):11618. doi: 10.3390/ijms241411618 (PMC10380639; doi:10.3390/ijms241411618)
Supplement: Supplementary file 1 [file ijms-24-11618-s001.zip › Supplementary Table 2 Mangiaterra et al., IJMS 2023.pdf]

Supplementary Table S2. Nucleotide substitutions in Toxin/Antitoxin modules of *P. aeruginosa* C30 and AR86 compared to the reference *P. aeruginosa* PAO1 sequences

***P. aeruginosa* strain**	**Locus tag**	**Protein family**	**Nucleotide substitutions**
C30	PA0124	relElike_domain	c.1110295C>T.
AR86			c.143585T>C; c.143806C>T.
C30	PA0125	relB	c.1111021T>C; c.1111461C>T; c.1111648T>C; c.1111655C>T; c.1111754A>G; c.1111837C>T; 1111857C>G; c.1112090T>C; c.1112134T>C.
AR86			c.143989T>G.
C30	PA0249	NULL	/
AR86			/
C30	PA0250	NULL	c.282695C>T; c.282697C>G.
AR86			c.282611G>T.
C30	PA1030	COG5654like_domain	c.1116674A>G; c.1116694C>G; c.1116868C>T; c.1116892C>T; c.1116920C>T; c.1116964C>T; c.1116984T>C; c.1117141A>G.
AR86			c.1116674A>G; c.1116694C>G; c.1116920C>T; C.1116984T>C; c.1117060C>G; c.1117133G>A; c.1117143C>G.
C30	PA1029	Xrelike_domain	c.1116285T>C.
AR86			c.1116171C>T; c.1116213_1116214insC; c.1116285T>C; c.1116332A>G.
C30	PA1227	HipAlike_domain	c.1329425G>A; c.1329461C>T; c.1329504T>C; c.1329533A>G; c.1329538C>A; c.1329593T>C; c.1329594G>A; c.1329983C>G.
AR86			c.1329214T>C; c.1329593T>C; c.1329594G>A; c.1329983C>G.
C30	PA1226	Xrelike_domain	c.1328552T>C; c.1328652T>C; c.1328667C>T; c.1328673A>T; c.1328835C>T; c.1328880C>A.
AR86			c.1328552T>C.
C30	PA1878	PINlike_domain	c.2048586A>G; c.2048810C>T; c.2048828A>C.
AR86			c.2048828A>C.
C30	PA1879	Xrelike_domain	/
AR86			/
C30	PA2083	NULL	c.2292197A>G; c.2292242G>C; c.2292253G>C; c.2292483T>C; c.2292486C>T; c.2292495A>G; c.2292567T>C; c.2292744C>T; c.2293046G>T.
AR86			c.2292197A>G; c.2292242G>C; c.2292253G>C; c.2292483T>C; c.2292486C>T; c.2292495A>G; c.2292567T>C; c.2292744C>T; c.2293046C>T.
C30	PA2082	NULL	c.2291277A>G; c.2291304G>A.
AR86			c.2291277A>G.

C30<br>AR86	PA2271	NULL	/<br>/
C30<br>AR86	PA2270	NULL	c. 2500625C>T; c.2500919G>C.<br>c.2500919G>T.
C30<br>AR86	PA2578	NULL	c.2914421A>G; c.2914432G>A; c.2914666T>C; c.2914703A>G.<br>c.2914421A>G; c.2914432G>A; c.291666T>C; c.2914794C>A.
C30<br>AR86	PA2577	NULL	c.2913930T>C; c.2914044G>A; c.2914056C>T.<br>/
C30<br>AR86	PA2693	NULL	c.3047574C>T.<br>/
C30<br>AR86	PA2692	NULL	c.3046513T>C.<br>c.3046513T>C.
C30<br>AR86	PA3248	NULL	c.3635449A>G.<br>c.3635139A>G; c.3635143C>G; c.3635260G>T; c.3635341C>T; c.3635395T>C; c.3635410T>C; c.3635449A>G.
C30<br>AR86	PA3249	NULL	c.3636069G>A; c.3636072A>G; c.3636102T>C.<br>c.3635614C>T; c.3635643G>A; c.3635703C>G; c.3635799T>C; c.3635883G>A; c.3636072A>G; c.3636102T>C.
C30<br>AR86	PA3270	GNATlike_domain	c.3659496C>T; c.3659529A>G.<br>c.3659496C>T; c.3659529A>G.
C30<br>AR86	PA3269	Xrelike_domain	c.3658257G>T; c.3658258A>G; c.3658269T>C; c.3658359C>T; c.3658569A>G; c.3658646C>T; c.3658653A>G; c.3658681T>C; c. 3658731G>A; c.3658761G>A; c.3658767C>T; c.3658815T>C.<br>c.3658681T>C; c.3658761G>A.
C30<br>AR86	PA4136	NULL	c.4625122C>T; c.4625343G>A; c.4625347G>C; c.4626034G>A; c.4626192G>A; c.4626193G>A; c.4626270C>G.<br>c.4625178C>T; c.4625344C>G; c.4626031G>A; c.4626270C>G.
C30<br>AR86	PA4135	NULL	c.4624911A>G; c.4624932G>A.<br>/
C30<br>AR86	PA4355	NULL	c.4883327G>A; c.4883507A>G.<br>/
C30<br>AR86	PA4354	NULL	c.4882226A>G; c.4882292C>T.<br>c.4882226A>G; c.4882292C>T.

/= no substitution found
